# How to Respond to Misinformation From the Anti-Vaccine Movement

**DOI:** 10.1177/00469580231155723

**Published:** 2023-02-17

**Authors:** Mike-Andrew Westhoff, Carsten Posovszky, Klaus-Michael Debatin

**Affiliations:** 1 University Medical Center Ulm, Ulm, Germany; 2 University-Children’s Hospital Zurich – Eleonorenstiftung, Zurich, Switzerland

**Keywords:** anti-vaccine movement, public health, literacy, vaccines, policy

## Abstract

Vaccines are doubtlessly one of the most crucial life-saving medical interventions to date. However, perplexingly, they court more public controversy than their objectively excellent safety profile warrants. While doubts about the safety of vaccines, as well as opposition to vaccine policies, can be traced back at least to the mid-19th century, the modern anti-vaccine movement has come in 3 distinct waves, or generations, each precipitating around distinct key events. Here, we describe the first 2 generations and trace the origins of an emerging third generation anti-vaccine movement. Currently, this third generation is an integral part of the larger anti-COVID movement and in this more libertarian environment propagates the idea of individualism superseding the responsibility for community health. We highlight the need for a better science education of the young, as well as the general public to further enhance overall science literacy and suggests strategies to achieve these goals.

**What do we already know about this topic?**

Although vaccines are among the most important medical interventions to be discovered, resistance to vaccination arose almost immediately. Since then organized anti-vaccine movements have come in 3 distinct but overlapping generations, that can be associated with certain (refuted) key beliefs: 1. Vaccines cause seizures, 2. Vaccines cause autism, and 3. Vaccines are not necessary to combat a perceived pandemic.

**How does your research contribute to the field?**

We show the origins of the third generation of the anti-vaccine movement that arose during the COVID pandemic and, using Germany as an example, show the underlying political currencies. Showing how arguments made against current vaccines have already been made by the preceding generations in the context of previous vaccines, we show a persistent anti-science attitude that prevails despite being repeatedly refuted.

**What are your research’s implications toward theory, practice, or policy?**

Our research demonstrates the generation-spanning use of misinformation and clearly indicates that children and adolescents, as well as their patents/legal guardians are targeted by the anti-vaccine movement. We highlight the need for better science education and improved media literacy and suggest appropriate action.

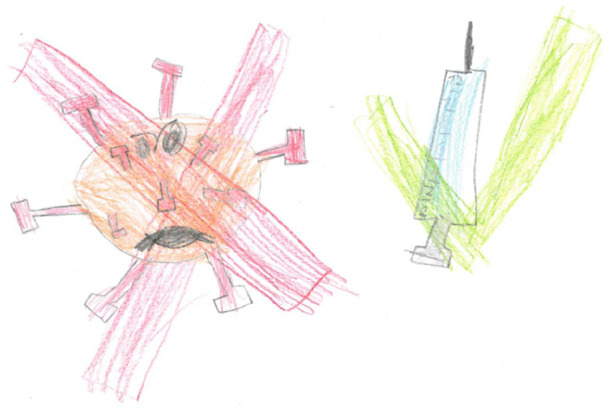


## Introduction: The Anti-Vaccine Movement—A Historical Outline

Aside from antibiotics it is difficult to think of any medical discovery or intervention that saved more lives than vaccines.^1^ This is also reflected in the high percentage of the global public strongly agreeing to the statements “vaccines are important,” “vaccines are effective” and “vaccines are safe.^2^ Although there can be little doubt that vaccines are safe by any objective assessment, like any medical procedure vaccines are not without risk. Rare and occasionally serious side effects can occur. This is a well-studied subject, with the National Library of Medicine’s PubMed web site listing more than 29 000 studies dealing with this subject and an excess of 1000 new annual studies being added since 2010.^3^

**Figure 1.**
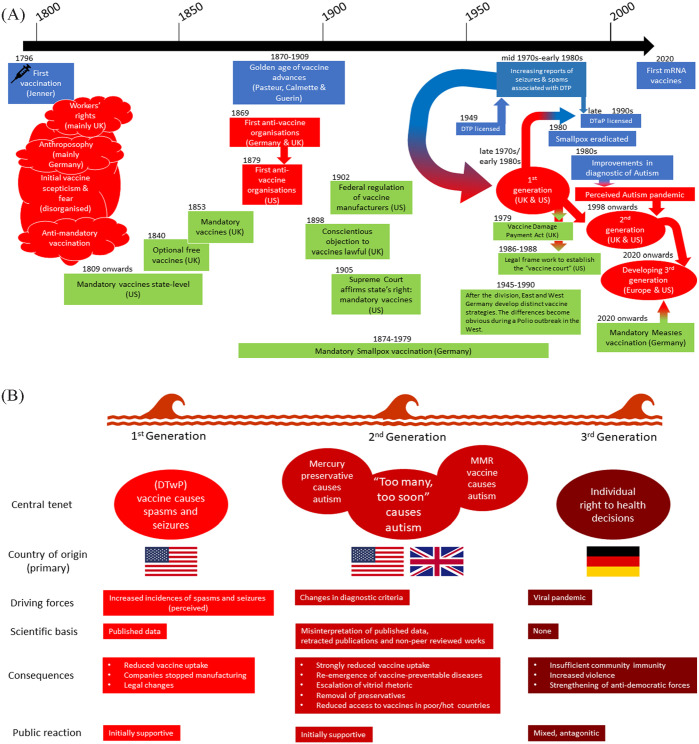
A brief history of vaccines and the anti-vaccine movement in 3 selected countries: (A) after Jenner’s initial success in 1789 unorganized opposite/fear to vaccination rapidly formed, in parallel—and surprisingly early—a legal frame work for mandatory vaccination was formulated in several countries. From the 1970s onward, networks formed and concentrated less on the individual rights of the patients, but started to question the very core idea of vaccination. Using 3 countries as examples, the UK, US, and Germany, we show the historical developments that led to the global reduction of several infectious diseases, as well as to the rise of organized anti-vaccine movements. Blue represents medical milestones, red highlights key developments in the anti-vaccine movement, while green illustrates the changing legal framework: (B) based on the available literature one can identify 3 distinct, but overlapping generations or waves within the continuous anti-vaccine movement.^7,8,10,13-21^ The first generation formed around the idea that (mainly) the diphtheria, tetanus, and pertussis (DTwP) vaccine was associated with spasms and seizures.^20^ Twenty years later a second-generation anti-vaccine movement formed around the scientifically untenable idea of an autism epidemy. This generation also attempts to highjack the scientific literature for propaganda purposes. The second generation also began to effectively cooped the media, described as “science by press release,” for example, when Andrew Wakefield claimed to have identified a link between the MMR (measles, mumps, and rubella) vaccine and autism in the now infamous and withdrawn 1996 study.^17^ Public multimedial speculations led to a loss in vaccine confidence, with the vaccine coverage ratio in England plummeting to 80% and measles being declared endemic again after 14 years for England and Wales in 2008.^19^ The third generation is currently still in the process of emerging and has yet to find a unifying global characterization. It currently appears as an integral part of a larger anti-science, health-freedom movement, as can also be seen in the “scientific” literature. The same author published papers claiming that for every 3 COVID cases prevented by vaccine 2 deaths occur and that children’s masks trap too-high concentrations of carbon dioxide, both now retracted.^22,23^ The individual generations, although characterized by distinct features, nevertheless strongly interact and overlap with each other. Robert F. Kennedy Jr., for example, one of the founding fathers of the second generation, appeared during a protest meeting against governmental COVID measures in Berlin, attacking both, the rollout of the 5 G-mobile network as a surveillance measure and Bill Gates.^24^

Nevertheless, already soon after Jenner’s popularization of vaccines in 1796, first concerns were raised against this new procedure.^4^ For example, a caricature by Gillray from 1802 showed vaccinated patients developing bovine features, eerily prescience of the transhumanism fears upon the introduction of RNA vaccines more than 200 years later.^5^ The initial anti-vaccine organizations appeared in parallel to the increased availability of vaccines and often contested the introduction of vaccine mandates, that is, were clearly associated with an anti-governmental stance (Figure 1A).^6^

This changed from the 1970s onward, when the focus shifted away from individual rights to questioning the very core idea of vaccination. The trigger for this shift was an increasing public concern regarding an association between whole-cell pertussis vaccine and neurological reactions, in particular seizures. This led to a decline in vaccine uptake and a rise of whooping cough incidences.^7,8^ Large scale studies have either failed to find an increased risk of seizures following vaccination, having focused on both diphtheria–tetanus–pertussis (DTP) and measles-mumps-rubella (MMR) vaccine, or could find alternative cause for most encephalopathy/encephalitis reported after vaccination. However, potentially casual links are still being reported from areas that continued to use the whole cell formulation.^8,9^ In the USA, perceived and rare real vaccine injuries were brought to court and juries frequently awarded substantial compensation to the claimants. This led to a sharp increase in manufacturing costs from 15 cents per dose DTP in 1980 to more than 14 Dollars in 1989, combined with the fact that vaccines accounted for only 15% of the revenue, but 40% of litigation cost of manufactory companies, the steady supply of vaccines was no longer guaranteed by the 1980s.^10^

This, in turn, led to *The National Vaccine Injury Compensation* Program (VICP or NVICP), a compensation program that both makes it easier for parents to be awarded compensation, if an injury is listed in the Vaccine Injury Table, and gives vaccine manufacturers more security, as accepting governmental compensation precludes additional court action.^53^ Work on the original legal draft was done by the American Academy of Pediatrics, as well as parents’ groups, notably Dissatisfied Parents Together (DPT).^11^DPT is the predecessor of the National Vaccine Information Center (NVIC) whose founder Barbara Lou Fisher must be considered a key figure of the anti-vaccine movement.^12^ Therefore, the resulting program makes a rare example of science-based medicine and anti-vaccine movement working to better the lives of children. These events can be seen as the first tightly organized generation or wave of the anti-vaccine movement (Figure 1B).

Twenty years later a second-generation anti-vaccine movement formed around the scientifically untenable idea of an autism epidemy caused by vaccines. Autism was initially thought to be a rare condition, early studies estimate a prevalence of 2 to 4 per 10 000, however, numbers published in the late 1980s and 1990s suggested an at least ten-fold higher incidence rate.^13^ However, together with an increased awareness for neurodiversity, this apparent rise in autism is mainly due to changes in diagnostic criteria. Autism now includes a wide variety of formally distinct characteristic summarized as autistic spectrum disorders (ASD), covering what used to be called Asperger’s Syndrome.^13^

Studies often find that the patient’s age of ASD diagnosis is around 3 years, that is, when most vaccines should be given.^25,26^ It is, therefore, not surprising that a perceived temporal relationship exists in a subset of children who did manifest autistic behavior soon after being vaccinated. Even in the absence of a feasible molecular model, it was doubtlessly correct to investigate a putative link between vaccines and autism; however, despite every effort being made, the scientific literature has failed to establish a causal relationship. The 2 best known proponents of this alleged vaccine-autism link are the American lawyer Robert Kennedy Jr. and the English physician Wakefield et al.^13,16^

In parallel to the rise of information technology, large international networks emerged around those 2 men which prove useful for the third generation, as a means to more efficient spread misinformation. The anti-vaccine movement is currently thriving on social media, where their existence and tactics were first described more than 10 years ago.^27^ A more recent British survey found that 50% of parents with children younger than 5 years regularly encounter negative “information” regarding vaccination.^28^ Interestingly, it has been shown that only a small number of people are responsible for the majority of vaccine misinformation on the internet, more precisely 12 people, dubbed “Disinformation Dozen” produce 65% vaccine misinformation on social media platforms.^29^ This suggests that the core anti-vaccine movement is not particularly large, but well versed in spreading misinformation. Yet, the much larger scientific community has so far failed to efficiently counter this misinformation.

## The Third-Generation Anti-Vaccine Movement (Not Only) in Germany—Back to the Roots

Despite a strong, institutionalized support for complementary and alternative medicine (CAM), the first 2 generations of the antivaccine-movement were rather minor events in Germany and left no lasting impression on the public’s consciousness. However, since the beginning of the new millenium repeated outbreaks of measles occurred, that frequently centered around non-immune anthroposophical communities.^30,31^ Here, we find philosophical similarities to the original often unorganized opposition to vaccination (Figure 1A). While the anthroposophical movement is not against vaccination as such, it “emphasises nature-based therapies that support the body’s innate healing wisdom” and is strictly against mandatory vaccines, focusing on the parental rights in the decision-making process.^30,32,33^ Nevertheless, in anthroposophical schools the vaccine uptake is frequently lower than in the general population.^34^ Despite this, it has been suggested that the generally low uptake in measles vaccine is due more to apathy than active opposition, so the German Ministry of Health launched a campaign in 2013 called “Deutschland sucht den Impfpass,” trying to encourage people to seek out and familiarize themselves with their vaccine documentation.^35,36^ Opposition to the campaign was mainly restricted to online forums, such as Facebook, where a group called “Deutschland verbrennt den Impfpass” (Germany burns the vaccine documentation) with as few as 3800 members predominantly warned of perceived damages caused by vaccination.^36^ In parallel to the outreach the German government looked, rather discreetly, into the possibility to re-introduce mandatory vaccination, but only specifically for measles, which finally occurred in March 2020.

The “Masernschutzgesetz” made vaccination against measles mandatory for substantial parts of the health care profession, asylum seekers and refugees, as well as school children. Neither vaccine hesitation of the anthroposophical communities, nor the preparation and introduction of the mandate were in the public focus, until the advent of the global COVID-19 pandemic and the world-wide resistance to governmental measures. The current demonstrations in Germany against the government’s management of the COVID-19 pandemic, the corona-denial offensive, consist of an eclectic mix of different, often contradictory movements which currently lack a strong leadership. Reports show that people worried about their future livelihood or concerned with the maintenance of democratic checks and balances are present as are followers of various conspiracy theories and those of authoritarian and anti-Semitic leanings.^37^ Demonstrations by the corona-denial offensive frequently feature attacks on Bill Gates and his vaccine campaign, as well as the fear of mandatory state interventions.^24^ Furthermore, rates of SARS-CoV-2 infection in Germany clearly correlated with areas where parties which are critical toward democratic institutions are strongly favored.^38^

**Figure 2.**
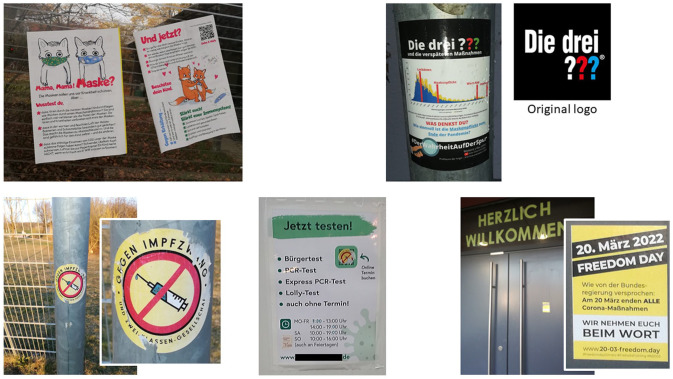
Examples of the corona-denial offensive, of which the third-generation anti-vaccine movement is an integral part, targeting children and parents. Top left: Unauthorized placards which had been put up at the bus stop in front of our hospital’s nursery. The notice on the left claims that masks do not prevent viral transmission, that the humid air under the masks is an ideal breeding ground for bacteria and fungi and that mask wearing leads to carbon dioxide poisoning. The notice on the right implies that COVID is similar to the common cold and strengthening a child’s immune system is sufficient to avoid serious health complication. The cute figures are obviously designed to catch the attention of children and their parents. Top right: On the left a note stuck to a lamppost is shown questioning the introduction of masks, warning-apps and vaccines toward the end of the COVID pandemic, defined as mid-April 2020. Note the design was deliberately chosen to mimic that of the highly successful detective stories “Die drei ???” (original logo reproduced with kind permission). “Die drei ???” stories, although also popular with adults who grew up with them, are primarily aimed at children from the age of 10 onward, while a similarly designed spin-off, called “Die drei ??? Kids,” is for younger children. This sticker is obviously aimed to entice children in that age bracket. Bottom left: In early 2022 the debate regarding mandatory vaccination against COVID grew more hostile, as certain sections of the population perceived the shifting stance of the German parliament as a breach of previously given assurances. Stickers against mandatory vaccination began to appear, again, often associated with places were families with children would gather. The example shown here is from a football pitch next to a popular playground. Bottom middle: A similar sticker placed over the QR code on a poster at the local test center, in essence making it impossible to make a timely appointment. Bottom right: The door of the aforementioned nursery, were briefly a sticker was placed without authorization. It depicts a veiled threat against the government, misinterpreting the shift in law that occurred on March, the 20^th^, 2022.

In addition to these highly political or philosophical arguments, we find many of the key untruths of the second-generation anti-vaccine movement are currently being re-used specifically geared toward SARS-CoV-2 (Table 1). Interestingly, we find, already early during the pandemic and prior to the licensing of a SARS-CoV-2 vaccine for children under 12, that parents and children were specifically targeted by the corona-denial offensive (Figure 2). More recently, we also see more aggressive attempts to politically declare the pandemic for over (Figure 2) and to sustain the anti-government and anti-media core of the anti-pandemic movement by shifting to new targets, for example the war in the Ukraine.^45,46^

**Table 1.** Anti-Vaccine Statements and Their COVID-19-Specific Variants.

Common anti-vaccine arguments (identified by Smith in 2017)^12^	COVID-specific variant
Vaccines are “toxic”	mRNA vaccines will permanently alter your DNA/make you transhuman^39^
Vaccines are a tool of “Big Pharma”	The pandemic is a ruse by big pharmaceutical companies to profiteer off a vaccine^40^
A child’s immune system is too immature to handle vaccines	Our immune system cannot handle the spike protein, which leads to infertility^41^
Natural immunity is better	COVID-19 is just a flu^42^
Vaccines have never been tested in a true “vaccinated versus unvaccinated” study	The FDA hasn’t fully authorized the Pfizer vaccine^43^
Diseases declined on their own due to improved hygiene and sanitation	Mask wearing, warn-app and vaccines were introduced at the end of the pandemic (see example in Figure 2)
Vaccines “shed”	Vaccinated people are just as likely’ to spread the coronavirus as unvaccinated^44^

## Evaluation of Current Responses to the Anti-Vaccine Movement

Currently, many proposals exist on how to deal with vaccine hesitancy, mainly focusing on better communication by scientists or a more proactive approach by governments, including the extension of vaccine mandates.^6,47,48^ While vaccine mandates for COVID have been shown to work, they remain associated with substantial ethical considerations and will do little to increase the public’s trust in science.^49,50^

Most approaches mainly target those members of the public unsettled by the circulating misinformation. Here, better communication and strategies to counter misinformation might alleviate insecurities, but most studies suggest that members of the anti-vaccine movements cannot be persuaded by evidence-based refutation of their talking points.^6^ In a recent interview forensic psychiatrist Heidi Kastner has suggested a current shift in societal believe systems, from the right to one’s own opinion to the right of one’s own facts and, thus, no dialog can be established as a commonly accepted basis in fact is missing.^51^

## Discussion: Improving Media and Science Literacy

The unique problem of today’s society is not a lack of information, as it was in the past, but that of disinformation hidden in the overabundance of information. Therefore, preparing the younger/next generation to critically evaluate information by scientific, evidence and fact-based thinking, that prevents being influenced by misinformation, is required. While this will not counter the third generation of the anti-vaccine movement or the broader anti-establishment attacks, the current pandemic is unlikely to be the last and the anti-vaccine sentiment is by far not the only anti-science movement.

Enhanced exposure to social media and messenger services, and, thus, the most potent source of misinformation, frequently begins around the age when children move from primary to secondary school and intensifies in the teenage years.^52^ It is important to have children and parents prepared for this new, additional source of misinformation. Good approaches can be found in student activities dedicated to these target groups. The Federal Representation of Medical Students in Germany e.V. (bvmd) organizes teddy bear clinics for pre-school children to take their cuddly toys to a medical examination and thus allows the children to familiarize themselves with medical procedures.^53^ In addition, the Verein für Impfaufklärung in Deutschland e.V. (Association for Immunisation Education in Germany), founded by medical students, provides basic immunological knowledge with vaccination education in secondary schools, among other things, and carries out a voluntary vaccination passport check.^54^ Another approach is “Forschungsferien” (research holidays) for staff children offered by several universities that combine teaching of scientific topics with a holiday camp. The problem here is the pre-selection of children from families with an already existing academic background, or for children/parents with a keen interest in medicine and/or science.

In contrast, we not only live in a society where anti-science sentiments have become increasingly popular, but are firmly entrenched in the medical community, for example, in form of anthroposophical medicine. Despite the origins of anthropomorphism that are closely associated with occultism and fascism and its clearly anti-science worldview, for example, ascribing the cause of the Spanish Flue to “the constellation of the outer planets, mediated through the sun” which “had a disruptive effect on the ‘head-chest-rhythm,’” this movement is often strongly supported by governmental regulators.^55,56^ Anthroposophical and homeopathic medicine is evaluated by the anthroposophical community itself in specific commissions of the Bundesinstitut für Arzneimittel und Medizinprodukte (BfArM, Federal Institute for Drugs and Medical Devices) and is explicitly excluded from needing to provide scientific evidence of effectiveness.^57-59^ The German parliament voted unanimously against implementing scientific standards, citing alternative medicine as “national achievement” and expression of “freedom.”^59^ Some health insurers even pay for non-standard treatment options, such as homeopathy,^60^ while support of vaccination among anthroposophical physicians has been lukewarm at best.^30,61^ In form of Waldorf education anthropomorphism is also entrenched in the (pre-)school system, where these schools have been associated with the spread of infectious, vaccine-preventable diseases and higher rates of personal belief exemptions to immunization requirements.^30,34,62^ However, it should also be stressed that this system of education is also leads to increased creativity in pupils, as well as significant benefits for preschool children’s development.^63,64^ Importantly, while anthroposophical schooling is associated with only moderate scientific achievements, pupils demonstrate an above average enjoyment and interest in science, that is, despite the unscientific basis of anthropomorphism, its inquiry-based science education actually has beneficial effects.^65^ Such an approach could also be used in a coordinated drive to increase better science education and improved media literacy.

We envision a much broader scientific-driven nationwide education initiative for pupils by teachers together with scientists, postgrad students, and students of different disciplines using evidence-based science to debunk misinformation. Importantly, to ensure maximal cooperation on all levels, we propose an approach that does not discourage particular worldviews, but provides a set of particular, age-appropriate tools and skills that allow the children to critically filter and evaluate information, put it into context and independently draw conclusions:

- Critical analysis of information/source evaluation, that is, an age-adjusted CRAAP test^66^
- Separation of facts and opinion
- Decoupling of information value and personal dislike or preference as well as emotional attachment the source of the information (confirmation biased/echo chamber)
- Understanding of experimental design and statistical analysis
- Adjustment of opinion based on new findings

Importantly, such a project, which can only be realized in cooperation with educators and child physiatrists, needs to cover these topics not in the context of a specific science-related example, as to avoid accusations of government-supported indoctrination; but inspired by the alleged “CSI-effect” and the anti-vaccine movements own strategy of misappropriating popular children’s detective stories (Figure 2), we could envision a project that covers aforementioned aspects in the fictional context of a crime story, such as the theft of a school/class mascot or the disappearance of milk money.

## Conclusion

While excellent advice how to deal with anti-vaccine sentiment on an individual basis and in a professional setting already exists, studies demonstrate that informed refutation of the latest argument against vaccination will be unlikely to change preconceived opinions.^6,67,68^ We propose an additional focus on what is to be done by the scientific community as educators and as parents. Here, in addition to the current suggestions of how to effectively counter the public’s insecurity due to anti-vaccine propaganda, such as supporting the spread of correct information in independent news outlets and use non-traditional routes to reach a broader audience,^69^ the scientific community also needs a more direct approach providing critical thinking tools and an age-appropriate understanding of scientific methods for pupils, as to prepare the next generation for future misinformation campaigns. This is in line with a 2018 survey by the prestigious Japanese newspaper, *The Asahi Shimbun*, which found that the key concern regarding the spreading of so-called fake news is that “news becomes unreliable,” while the most important countermeasure to be implemented was identified as “literacy education.”^70^
